# Perceived physical literacy and subjective wellbeing in university students: the indirect association via autonomous sports motivation

**DOI:** 10.3389/fpsyg.2026.1860366

**Published:** 2026-07-10

**Authors:** Xiongwei Xiang

**Affiliations:** College of Physical Education, Hunan Normal University, Changsha, Hunan, China

**Keywords:** autonomous sports motivation, physical literacy, self-determination theory, subjective wellbeing, university students

## Abstract

**Objective:**

This cross-sectional study examines the association between Perceived Physical Literacy (PPLI) and Subjective Wellbeing (SWB) among Chinese university students, exploring the indirect association of Autonomous Sports Motivation.

**Methods:**

A cross-sectional survey sampled 858 students (*M* = 19.15, *SD* = 1.07) from ten different mainland Chinese universities. Validated instruments measured PPLI, the Behavioral Regulation in Exercise Questionnaire (BREQ-3) for motivation, and a composite scale for SWB. A higher-order Confirmatory Factor Analysis (CFA) was conducted to ensure the measurement model for the Relative Autonomy Index (RAI) was methodologically consistent with the structural model. Structural Equation Modeling with bias-corrected Bootstrap procedures tested the indirect effects, controlling for gender, age, and BMI.

**Results:**

A significant direct positive association was found between PPLI and SWB (β = 0.27, *p* < 0.001). Crucially, Autonomous Sports Motivation (Relative Autonomy Index) served as a significant statistical mediator (indirect effect = 0.23, 95% CI [0.186, 0.281], *p* < 0.001). Higher physical literacy was associated with higher-quality autonomous motivation (β = 0.43), which in turn was associated with greater wellbeing (β = 0.42). The variables collectively accounted for 35% of the variance in SWB.

**Conclusion:**

Although the cross-sectional design limits causal inferences, the findings underscore an indirect association between university physical education perceptions and psychological health. The results suggest that promoting physical literacy may relate to higher autonomous motivation, which is associated with better wellbeing. These results advocate for curriculum designs that prioritize “proactive health” over “passive participation.”

## 1 Introduction

### 1.1 Research background: seeking the source of wellbeing in physical education

In the global landscape of higher education, contemporary university students face a growing paradox: on one hand, they enjoy unprecedented material abundance and informational convenience; on the other, their mental health status continues to raise red flags. Multiple reports from the World Health Organization (WHO) indicate that psychological distress such as anxiety and depression is prevalent among youth, becoming a global public health challenge that cannot be ignored (Auerbach et al., 2018). More recent evidence from the post-COVID-19 era indicates a significant increase in these challenges among higher education students (Chautrakarn et al., 2024). Against this backdrop, Subjective Wellbeing (SWB), a comprehensive psychological indicator measuring an individual’s overall quality of life and emotional experience, has been elevated to unprecedented importance. It not only pertains to an individual’s life satisfaction and positive affect but is also considered a key predictor of personal achievement, social adaptation, and long-term health (Moussa-Chamari et al., 2024).

How can we find a universal, efficient, and cost-effective path within the institutionalized education system to cultivate the wellbeing of university students? Physical activity, as one of the oldest forms of human vitality, has been widely proven to promote mental health (Herbert, 2022; Rodríguez-Romo et al., 2022; Ahsan and Abualait, 2025). However, compared to spontaneous and sporadic individual exercise, university Physical Education (PE) classes offer a unique, structured intervention setting. With broad coverage throughout a student’s critical developmental period, it is theoretically an ideal platform for promoting the physical and mental health of all students (Donnelly et al., 2024; Talapko et al., 2021). Yet, practical observations suggest that the effectiveness of PE classes is not always as expected. Many students view PE as a mandatory requirement lacking intrinsic enthusiasm, and rarely engage in physical activity voluntarily after the course ends (Haug et al., 2023). This phenomenon of “knowing is easy, but doing is hard” leads us to ponder a deeper question: What should PE classes “teach” and “how should they teach it” to potentially foster psychological health? The starting point of this study is to attempt to answer this question.

### 1.2 Physical literacy: personal capital beyond skills

Traditional physical education often overemphasizes the mastery of motor skills and competitive achievement, a model that is often associated with feelings of frustration and alienation among students with poorer skills (Lunde et al., 2023). In recent years, a more inclusive and forward-looking concept—Physical Literacy (PL)—is gradually reshaping our understanding of the goals of physical education. According to the seminal definition by the International Physical Literacy Association, physical literacy is the motivation, confidence, physical competence, knowledge and understanding to value and take responsibility for engagement in physical activities for life (Whitehead, 2010). This concept has been widely developed and validated through various scales in multiple countries and regions (Sum et al., 2016; Ma et al., 2020; Liu et al., 2022; Mendoza-Muñoz et al., 2023; Gandrieau et al., 2023).

The core of this definition lies in its holistic and internally consistent nature. It no longer views individuals as mere executors of motor techniques but emphasizes the organic integration of four dimensions: motivation, confidence, physical competence, and knowledge and understanding (Luo et al., 2022). It is important to distinguish perceived physical literacy from autonomous motivation; while both constructs encompass motivational elements, perceived physical literacy reflects a holistic sense of competence and readiness for an active life, whereas autonomous motivation specifically describes the self-determined quality of the psychological drive behind specific physical behaviors.

From a psychological perspective, higher levels of perceived physical literacy are theoretically linked to a more positive self-concept: when students perceive higher levels of their physical competence, it correlates with an increased sense of self-efficacy; meanwhile, possessing greater knowledge about physical activity is associated with a stronger sense of control over their lives (Hu and Wang, 2026). However, can this personal capital derived from the physical level be associated with a psychological experience of wellbeing? In recent years, preliminary studies have suggested a positive association between physical literacy and mental health (Ma et al., 2021; Melby et al., 2023; Souza-Lima et al., 2025), and this link has been observed across different cultural contexts (Jiang et al., 2024). During the critical stage of early adulthood, what is the exact nature of the associational link between perceived physical literacy and the broader life satisfaction of university students? Based on this, the present study attempts to test the following hypothesis:

*H1*: There is a significant positive association between university students’ Perceived Physical Literacy (PPLI) and their Subjective Wellbeing (SWB).

### 1.3 The indirect association via autonomous sports motivation: a self-determination theory perspective

Although the associational link between physical literacy and wellbeing is emerging, its underlying statistical pathway remains to be fully elucidated. Here, Self-Determination Theory (SDT; Deci and Ryan, 2000) provides a sophisticated explanatory framework. SDT posits that when an environment satisfies an individual’s basic psychological needs for autonomy, competence, and relatedness, their behavioral motivation is more likely to be perceived from passive “controlled motivation” to high-quality “autonomous motivation” (Vallerand et al., 2008). Numerous meta-analyses have shown that SDT-based interventions are associated with higher motivational quality and mental health outcomes in the health domain (Ntoumanis et al., 2021), an effect also widely confirmed in the context of physical exercise among adolescents and university students (de Oliveira Barbosa et al., 2024; Qi et al., 2024).

Placing this theory within the context of PE classes, the physical literacy and the quality of motivation show a high degree of theoretical resonance (Tilga et al., 2023; Llanos-Muñoz et al., 2023). A course that emphasizes physical literacy often provides an environment conceptually aligned with these three psychological needs. The perception of competence is consistent with the need for competence, while the establishment of confidence provides the psychological prerequisite for autonomously choosing physical activities (Hutmacher et al., 2022). This leads to two specific scientific questions: First, is the level of perceived physical literacy associated with higher levels of internalization of students’ autonomous sports motivation? Second, is this autonomous motivation associated with life satisfaction and subjective vitality? Based on the theoretical reasoning above, we propose the following hypotheses:

*H2*: Perceived Physical Literacy (PPLI) is significantly and positively associated with students’ autonomous sports motivation (as indicated by RAI).

*H3*: Autonomous sports motivation (RAI) is significantly and positively associated with students’ Subjective Wellbeing (SWB).

If we further examine this logical chain, we might find that physical literacy may not directly be linked to wellbeing. Perhaps it relates to wellbeing via an individual’s motivation for participation, and it is this high-quality motivation that is associated with lasting positive psychological experiences (Jankauskiene et al., 2022). Therefore, does autonomous sports motivation play an indirect role between perceived physical literacy and subjective wellbeing? To clarify this statistical pathway, this study proposes:

*H4*: Autonomous sports motivation is a significant statistical mediator in the relationship between perceived physical literacy and subjective wellbeing.

### 1.4 Theoretical framework and purpose of this study

**FIGURE 1:**
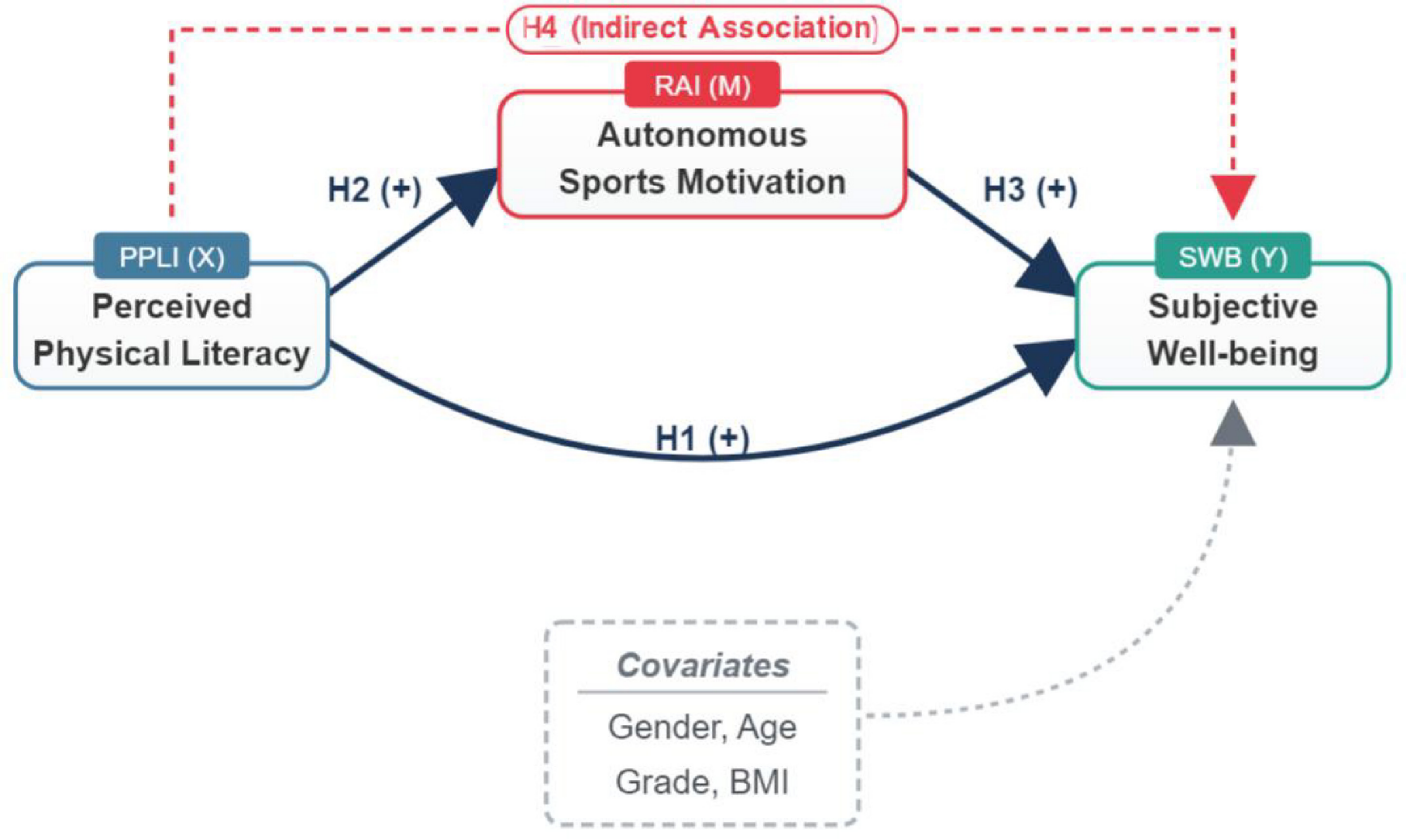
Theoretical model diagram.

Synthesizing the discussion of the above questions, this study aims to construct a theoretical model examining the links between physical literacy, autonomous sports motivation, and subjective wellbeing, as shown in Figure 1. This study makes three incremental contributions to the literature. First, unlike previous research that examined PL primarily in relation to physical health outcomes (Sum et al., 2016; Luo et al., 2022), we position PL as a psychological antecedent within the SDT framework to test the quality of motivation. Second, expanding on the behavioral pathways examined by Melby et al. (2023)—who demonstrated how PL relates to wellbeing through sport and exercise participation—we specifically investigate a motivational link, arguing that the quality of psychological drive (RAI) is as relevant to wellbeing as exercise volume. Third, building on the evidence provided by Ma et al. (2021) regarding PL and mental health in Chinese students, our multi-group analysis addresses whether these associations extend to sedentary or exercise-resistant groups (Donnelly et al., 2024), providing direct evidence for the universal importance of PL cultivation. We are not content with merely verifying the universal conclusion that ‘exercise is good for health.’ Instead, we attempt to delve deeper into how, in the context of Chinese university physical education, perceived competence is associated with motivation and wellbeing (Sauri et al., 2025).

The theoretical aim of this study is to examine the associations between the “physical body” and “psychological health.” By organically combining the theoretical frameworks of Physical Literacy Theory and Self-Determination Theory (Abdulla et al., 2022; Sarfo et al., 2023), we not only focus on the associations with physical literacy but also strive to investigate these relationships through a motivational lens. This will help clarify the potential role of physical literacy in fostering students’ wellbeing from being “skill-oriented” to “literacy-oriented” (Liu et al., 2024; Gao et al., 2024).

## 2 Materials and methods

### 2.1 Participants and procedure

The data for this study were collected through a cross-sectional questionnaire survey targeting university students in mainland China. Participants were recruited from ten different universities across diverse academic disciplines (e.g., liberal arts, science, and engineering) located in central and southern China to enhance the representativeness of the sample. We used the online questionnaire platform “the Wenjuanxing platform (a professional online survey platform in China)” and employed a combination of convenience sampling and snowball sampling to collect data from September to November 2025. To ensure data uniqueness and prevent duplicate responses, the survey platform’s restriction setting was enabled to allow only one submission per IP address and device.

The protocol was approved by the Institutional Review Board (IRB) of the College of Physical Education at Hunan Normal University (Approved on September 5, 2025). The committee granted an ethical exemption for this study because it involved anonymous procedures with no risk to participants. All potential participants were presented with a detailed informed consent form on the first page of the questionnaire, which clearly stated the research purpose, measures for ensuring data anonymity, and their unconditional right to withdraw from the study at any time. The system was configured so that only individuals aged 18 or above who clicked a mandatory electronic checkbox “I Agree to participate” could proceed to the formal questionnaire.

During the data preprocessing stage, we adopted a rigorous approach to ensure data quality. The initially collected 960 questionnaires underwent three rounds of screening: first, listwise deletion was used to remove samples with systematic missing values in the core scale sections; second, an embedded “attention check question” (e.g., “For this question, please select ‘Disagree slightly”’) was used to filter out invalid questionnaires from participants who clearly did not answer seriously; finally, we used the *Z*-score method (threshold set at |*Z*| > 3) to test the calculated core variables (e.g., BMI, total scores of each scale) to identify and handle extreme outliers. In total, 102 cases were excluded (including 42 failed attention checks, 35 with missing data, and 25 outliers). After this cleaning process, a final valid sample of 858 was retained, resulting in an effective response rate of 89.4%.

The demographic characteristics of the 858 participants included in the final analysis are shown in Table 1. The sample consisted of 455 males (53.0%) and 403 females (47.0%), showing a relatively balanced gender distribution. The average age of the participants was 19.15 years (*SD* = 1.07), with an age range of 18–25. In terms of grade distribution, freshmen and sophomores constituted the majority of the sample (38.8 and 36.7%, respectively), which is consistent with the fact that university PE is often a compulsory course for lower-year students. The participants’ average Body Mass Index (BMI) was 21.88 kg/m^2^ (*SD* = 3.43), falling within the healthy weight range.

**TABLE 1:** Demographic characteristics of participants (*N* = 858).

Characteristic	Category	N	Percentage (%)
Gender	Male	455	53.0
	Female	403	47.0
Grade	Freshman	333	38.8
	Sophomore	315	36.7
	Junior	123	14.3
	Senior	67	7.8
	Postgraduate	20	2.3
Age	Mean ± SD	19.15 ± 1.07	–
BMI (kg/m^2^)	Mean ± SD	21.88 ± 3.43	–

### 2.2 Measures

The core constructs of this study were measured using established scales with demonstrated psychometric properties in Chinese university populations, ensuring the reliability and validity of the measurements. During the data entry phase, all reverse-scored items (such as the amotivation items in BREQ-3) were recoded according to scoring rules. To ensure linguistic clarity, the final survey items were cross-checked with the original English versions, which are detailed in Supplementary Appendix 1.

#### 2.2.1 Perceived physical literacy (PPLI)

We used the short-form Perceived Physical Literacy Instrument (PPLI), which was originally developed by Sum et al. (2016) and later validated for Chinese undergraduates by Ma et al. (2020). The 8-item version was selected due to its superior fit and reduced respondent burden while maintaining the core theoretical dimensions of the original scale (Sum et al., 2016; Ma et al., 2020). This instrument has since been widely applied to various adult populations after cross-cultural adaptation (Gandrieau et al., 2023; Liu et al., 2022; Mendoza-Muñoz et al., 2023). The scale assesses students’ subjective perception across three dimensions: physical competence, knowledge and understanding, and self-expression. An example item is, “I clearly understand the specific effects of different types of physical activities on maintaining physical and mental health.” The scale uses a 5-point Likert scoring method, from 1 (Strongly disagree) to 5 (Strongly agree). The internal consistency reliability (Cronbach’s α) in the present study was 0.840.

#### 2.2.2 Autonomous sports motivation and relative autonomy index (RAI)

To measure the type of students’ motivation for participating in physical activities, this study referenced the Behavioral Regulation in Exercise Questionnaire (BREQ-3) developed by Markland and Tobin (2004) and Wilson et al. (2006). We selected 11 items that have previously demonstrated high factor loadings in similar student samples to represent the six dimensions of the SDT continuum; the complete list of items is provided in Supplementary Appendix 1. All items were rated on a 5-point Likert scale (1 = Not at all true for me, 5 = Very true for me).

To represent the self-determination index of motivation as a single variable in the structural analysis, we calculated the “Relative Autonomy Index (RAI)” (Vallerand et al., 2008). Following standard SDT scoring procedures, the RAI calculation formula is presented as follows formula (1):


$$\mathrm{RAI}=3\,M_{\mathrm{intrinsic}}+2\,M_{\mathrm{integrated}}+1\,M_{\mathrm{identified}}-1\,M_{\mathrm{introjected}}$$ (1)

$$-2\,M_{\mathrm{external}}-3\,M_{\mathrm{amotivation}}$$

where *M* represents the mean score of the retained items for each motivational regulation subscale. A higher RAI score indicates higher-quality self-determined motivation for physical activity. In the structural model, the RAI was treated as an observed weighted composite variable. It should be explicitly noted that while the main CFA (reported in section 3.2) exclusively utilized the 6 highest-loading autonomous motivation items to conceptualize the latent construct for optimal model parsimony, the calculation of the overall RAI incorporated all 11 retained items (Q20-Q30). Because the RAI includes both autonomous and controlled forms of regulation, it should be interpreted as an index of relative self-determination rather than as a latent factor identical to the AUTO_MOT construct. Detailed scoring keys, specific questionnaire items, and their weighted integration for research reproducibility are provided in Supplementary Appendix 2.

#### 2.2.3 Subjective wellbeing (SWB)

Following the comprehensive definition of wellbeing in psychology, we measured it from both cognitive and affective dimensions. The cognitive dimension was assessed using the widely validated Satisfaction with Life Scale (SWLS) developed by Diener et al. (1985), which includes 4 items (e.g., “In most ways my life is close to my ideal”). The affective dimension was measured using the short-form Subjective Vitality Scale (SVS), developed by Ryan and Frederick (1997) and further validated by Bostic et al. (2000), which includes 4 items (e.g., “Lately, I have been feeling full of energy”). Instead of focusing on the absence of negative emotions, we intentionally selected subjective vitality as the affective indicator because it more precisely captures the energetic arousal and psychological gains unique to physical activity contexts. All 8 items from both subscales used a uniform 7-point Likert scale (1 = Strongly disagree, 7 = Strongly agree), and the average score of all items was used as an indicator of an individual’s overall subjective wellbeing. Combining these cognitive and affective dimensions yields a holistic index of psychological flourishing that is well-suited for sports science research. The Cronbach’s α for this composite scale in the present study was 0.871.

#### 2.2.4 Control variables

Considering that individuals’ demographic and physiological characteristics could potentially be related to their willingness to participate in physical activity and their psychological state, this study included participants’ gender (coded as 1 = male, 2 = female), age, grade, subjective health status, frequency of extracurricular exercise, and calculated BMI as covariates in the subsequent statistical analysis models. This was done to more purely examine the relationships among the core variables.

### 2.3 Data analysis

All data processing in this study was conducted using SPSS 26.0 and AMOS 24.0 statistical software, with the significance threshold for hypothesis testing set at α = 0.05.

In the pre-modeling data verification stage, common method bias (CMB) was assessed using both Harman’s single-factor test and the common latent factor (CLF) approach to more rigorously confirm that variance was not primarily attributable to the measurement method (Podsakoff et al., 2003). Descriptive statistics, independent samples *t*-tests, and one-way analysis of variance (ANOVA) were primarily used to present the basic distribution of core variables and potential group differences, while Pearson correlation analysis provided preliminary evidence of statistical associations.

The core model validation followed the “two-step” structural equation modeling (SEM) approach advocated by Anderson and Gerbing (1988). First, confirmatory factor analysis (CFA) was used to assess the measurement model. Specifically, to ensure the structural consistency of the self-determination continuum, a higher-order CFA was performed to validate the hierarchical organization of the six motivation facets, providing a sound latent foundation for the subsequent use of the RAI. The structural model included participants’ gender, age, and BMI as control variables to isolate the unique associations of the independent variables with the dependent variable. To test the indirect associations of autonomous motivation, a maximum likelihood estimation method combined with a bias-corrected non-parametric Bootstrap procedure (with 5,000 resamples) was employed. A significant indirect effect was confirmed if the 95% confidence interval did not cross zero.

To test the robustness of the model, a multi-group SEM was conducted. Based on the WHO general recommendations for physical activity (which typically associate sufficient health benefits with ≥ 3 sessions/week), the sample was dichotomized into two practically meaningful groups: a “Low-to-Moderate Frequency” group (exercising ≤ 2 times/week, encompassing those lacking sufficient regular active habits) and a “High Frequency” group (exercising ≥ 3 times/week). Prior to comparing the structural path coefficients across these divergent behavioral groups, multigroup measurement invariance (configural, metric) was assessed to ensure the underlying factor structure remained invariant.

## 3 Results

### 3.1 Common method variance and data preprocessing tests

Before the formal analysis, a preliminary examination of data quality was a crucial step to ensure the reliability of subsequent conclusions. We first conducted Harman’s single-factor test on all Likert scale items. The results showed that, without rotation, the first principal component extracted by exploratory factor analysis explained only 29.8% of the total variance, a value well below the 40% threshold. To further address common method bias (CMB) more rigorously, we also employed the common latent factor (CLF) approach. Comparing the model with and without the CLF, the difference in standardized path loadings was less than 0.20, reinforcing that common method variance was not a major threat in this study.

Additionally, normality tests for the main constructs (PPLI, RAI, SWB) showed that their skewness and kurtosis values all fell within the ideal range of [−1, 1]. Furthermore, potential multicollinearity was assessed through variance inflation factors (VIF). All VIF values were below 3.0, indicating that multicollinearity did not pose a significant risk to the structural model.

### 3.2 Reliability and validity of the measurement model

To test the measurement quality of the core constructs in this study, we built a confirmatory factor analysis (CFA) model with three latent variables: Perceived Physical Literacy (PPLI), Autonomous Motivation (AUTO_MOT), and Subjective Wellbeing (SWB). To ensure the structural integrity of the motivational construct, we first conducted a higher-order CFA. This step validated that the distinct behavioral regulations successfully converged onto a single higher-order factor (χ^2^/*df* = 2.75, CFI = 0.944, TLI = 0.938, RMSEA = 0.044, SRMR = 0.045), thereby providing empirical justification for the use of the Relative Autonomy Index (RAI) in the final structural analysis.

**FIGURE 2:**
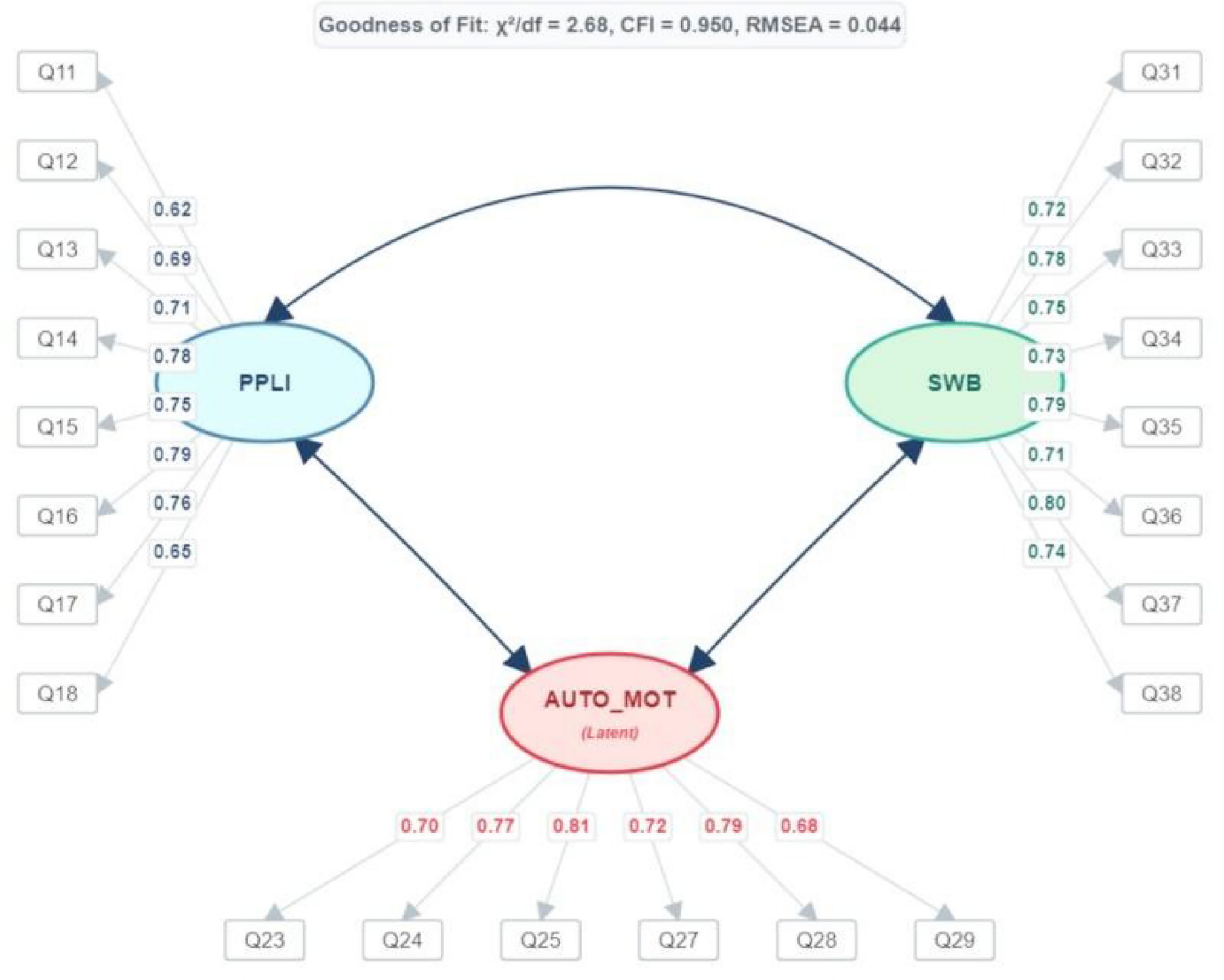
CFA measurement model diagram.

The structure of the CFA measurement model and its standardized path loadings are shown in Figure 2. The model’s fit indices were good, with all indicators meeting acceptable academic standards (χ^2^/*df* = 2.68, CFI = 0.950, TLI = 0.944, RMSEA = 0.044, SRMR = 0.041), indicating that our hypothesized measurement model structure fit the collected data well.

As shown in Table 2, the results of the reliability and validity tests were also satisfactory. In terms of internal consistency reliability, the Cronbach’s Alpha coefficients for the three core constructs were 0.840 (PPLI), 0.852 (Autonomous Motivation), and 0.871 (SWB). The composite reliability (CR) values were also all above 0.85, further confirming the reliability of the measurements. In terms of convergent validity, the standardized factor loadings of all observed items on their corresponding latent variables were above 0.60 and statistically significant (*p* < 0.001). Item Q26 was omitted from the initial motivation scale because its loading was below 0.40, likely due to cultural nuances in how negative feelings about exercise are perceived in the Chinese context. Furthermore, the average variance extracted (AVE) for each latent variable exceeded the 0.5 threshold.

**TABLE 2:** Reliability and validity indicators of the Main CFA measurement model.

Construct/latent variable	Item	λ	Cronbach’s α	CR	AVE
PPLI	Q11-18	0.62–0.79	0.840	0.896	0.520
AUTO_MOT	Q23, Q24, Q25, Q27, Q28, Q29	0.68–0.81	0.852	0.883	0.558
SWB	Q31-38	0.71–0.80	0.871	0.913	0.566

*N* = 858. CR, Composite Reliability; AVE, Average Variance Extracted. PPLI, Perceived Physical Literacy; AUTO_MOT, Autonomous Motivation; SWB, Subjective Wellbeing. All factor loadings are significant at *p* < 0.001.

This measurement model confirms that the observed indicators accurately reflect their respective latent constructs, allowing the RAI to be treated as a robust indicator of the total motivational profile in the structural analysis. The full set of 11 items was retained for the calculation of the Relative Autonomy Index (RAI) (Markland and Tobin, 2004; Wilson et al., 2006); the specific items and their corresponding SDT regulation weights are detailed in Supplementary Appendix 2.

### 3.3 Descriptive statistics, group differences, and correlation analysis

Table 3 presents the means, standard deviations, and Pearson correlation matrix of the main variables in this study. In terms of mean values, the students’ perceived physical literacy score was 3.45 (*SD* = 0.84), slightly above the midpoint of 3 on the 5-point scale, suggesting that the sample as a whole had a moderately positive self-assessment of their physical literacy. The average score for subjective wellbeing was 4.47 (*SD* = 1.08), also at a moderately positive level on the 7-point scale. These results are generally consistent with findings from similar studies (Moussa-Chamari et al., 2024; Rodríguez-Romo et al., 2022).

**TABLE 3:** Descriptive statistics, correlation matrix, and discriminant validity of major variables.

Variable	*M*	*SD*	1	2	3	4
1. PPLI	3.45	0.84	(0.721)	–	(0.752)	(0.747)
2. RAI	6.60	7.61	0.43**			
3. SWB	4.47	1.08	0.45**	0.53**		
4. AUTO_MOT	3.52	0.90	0.46**	0.81**	0.50**	
5. BMI	21.88	3.43	−0.04	−0.00	0.03	−0.00

*N* = 858. *M*, Mean; *SD*, standard deviation; PPLI, Perceived Physical Literacy; RAI, Relative Autonomy Index; SWB, Subjective Wellbeing; AUTO_MOT, Autonomous Motivation latent variable; BMI, Body Mass Index. The values in parentheses on the diagonal are the square roots of the AVE for each latent variable. ** *p* < 0.01.

The results of the correlation analysis provide initial support for our hypothesized model. The data clearly show a moderately strong positive correlation between perceived physical literacy and autonomous sports motivation (RAI) (*r* = 0.43, *p* < 0.001). This association is particularly crucial as it intuitively reflects that when students feel more “able, confident, and knowledgeable” in physical activities, the quality of their motivation to participate is correspondingly higher. This aligns well with the findings of Hutmacher et al. (2022) and de Oliveira Barbosa et al. (2024) in similar research. Furthermore, both variables showed a significant positive association with subjective wellbeing (PPLI: *r* = 0.45, *p* < 0.001; RAI: *r* = 0.53, *p* < 0.001). Notably, all Pearson correlation coefficients were below the 0.70 threshold, providing further empirical evidence alongside the VIF results (as reported in section 3.1) that multicollinearity does not pose a threat to the structural model. Additionally, the correlation between RAI and the latent AUTO_MOT variable was strong (*r* = 0.81, *p* < 0.01), reinforcing the methodological consistency of using RAI as a high-density indicator of motivational quality. An interesting detail is that the correlation coefficient between RAI and SWB was slightly higher than that between PPLI and SWB, which preliminarily suggests that motivation might be a psychological variable more closely related to the experience of wellbeing (Jankauskiene et al., 2022). Additionally, the discriminant validity test, as shown in the parentheses on the diagonal of Table 3, indicates that the square root of each construct’s AVE is greater than its correlation coefficients with other constructs, satisfying the Fornell-Larcker criterion and demonstrating the independence of the constructs.

**FIGURE 3:**
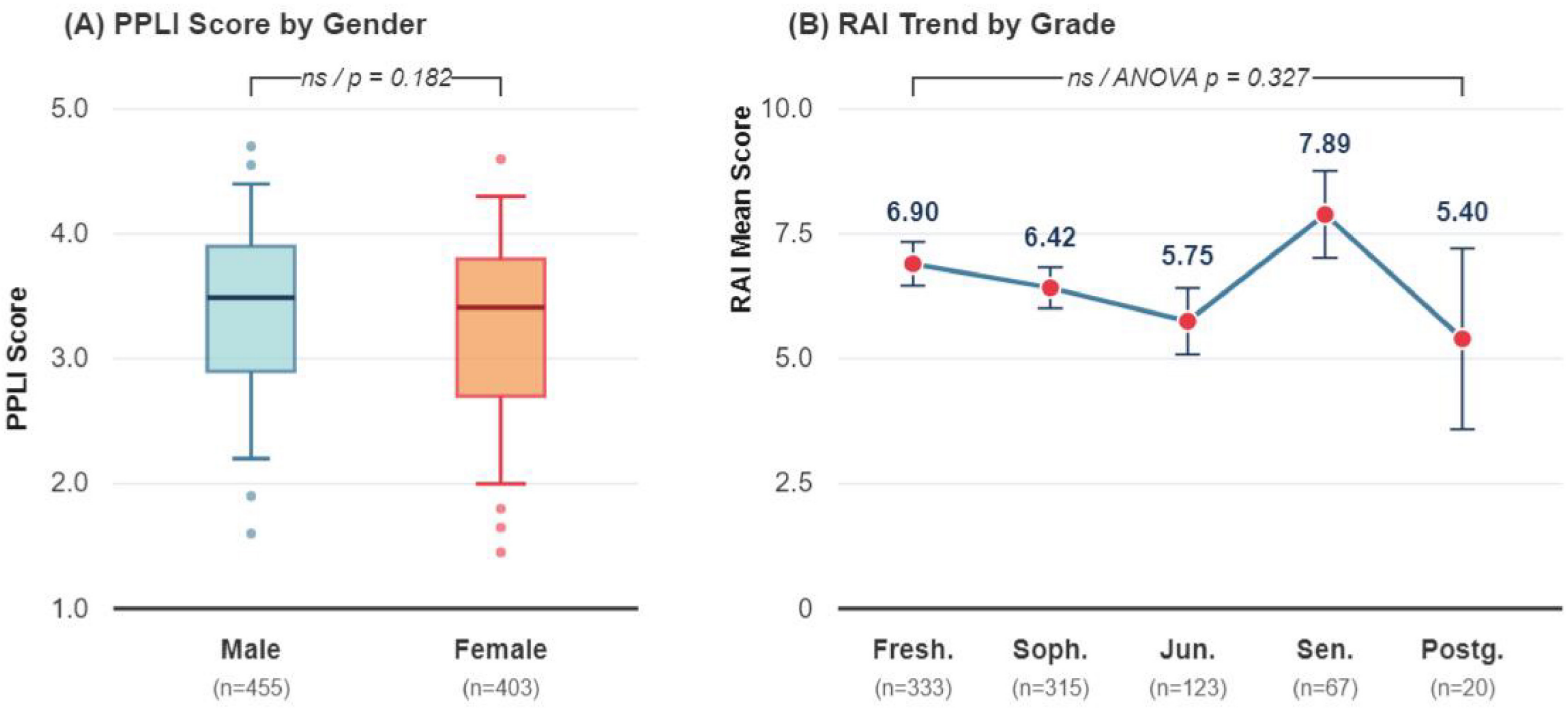
Group difference analysis of core psychological variables in university students. **(A)** Boxplot comparing the distribution and mean of Perceived Physical Literacy (PPLI) between male and female students. **(B)** Trend graph of the mean and standard error of Autonomous Sports Motivation (RAI) scores across different grades. No group comparisons reached statistical significance (ns).

Further group difference tests on demographic variables were conducted, with results shown in Figure 3 and Table 4. On the gender dimension (see Figure 3A), an independent samples *t*-test showed that males had a slightly higher average score on perceived physical literacy (PPLI) (*M* = 3.49, *SD* = 0.85) than females (*M* = 3.41, *SD* = 0.82), but this difference did not reach statistical significance [*t*(856) = 1.34, *p* = 0.182, Cohen’s *d* = 0.09]. On the grade dimension (see Figure 3B), an analysis of variance (ANOVA) showed no significant main effect of grade on autonomous sports motivation (RAI) among students [*F*(4, 853) = 1.16, *p* = 0.327, η^2^ = 0.005]. The small effect sizes observed (*d* < 0.2, η^2^< 0.01) suggest that the core psychological constructs remain relatively stable across gender and academic stages within this population.

**TABLE 4:** Group difference tests for core variables across demographic subgroups.

Variable	Grouping	Group	N	M	SD	Test statistic	*p*-value	Effect size
PPLI	Gender	Male	455	3.49	0.85	*t*(856) = 1.34	0.182	Cohen’s *d* = 0.09
		Female	403	3.41	0.82			
RAI	Grade	Freshman	333	6.90	8.04	*F*(4, 853) = 1.16	0.327	η^2^ = 0.005
		Sophomore	315	6.42	7.29			
		Junior	123	5.75	7.36			
		Senior	67	7.89	7.14			
		Postgraduate	20	5.40	8.10			

PPLI, Perceived Physical Literacy; RAI, Relative Autonomy Index. Effect size Cohen’s *d* is for *t*-test, η^2^ (Eta-squared) is for ANOVA.

This lack of significant differences at the full-sample level suggests that the cultivation of physical literacy and the internalization of motivation have universal educational intervention value for all university students, regardless of gender or educational stage (Melby et al., 2023).

### 3.4 Structural model and indirect association analysis

To test the core hypothesis of this study—the mediating role of autonomous sports motivation in the relationship between physical literacy and subjective wellbeing—we constructed a structural equation model. After controlling for the effects of gender, age, and BMI, the final structural model showed a good fit with the data (χ^2^ /*df* = 2.88, CFI = 0.941, TLI = 0.935, RMSEA = 0.046, SRMR = 0.043), indicating that our theoretical model effectively explained the relationships among the variables in the sample data (see Table 5).

**TABLE 5:** Fit indices of the structural equation model.

Fit index	Recommended criteria	Model value	Conclusion
χ^2^/*df* (Chi-square to degrees of freedom ratio)	<3.0	2.88	Good
CFI (Comparative Fit Index)	>0.90	0.941	Good
TLI (Tucker-Lewis Index)	>0.90	0.935	Good
RMSEA (Root Mean Square Error of Approximation)	<0.08	0.046	Excellent
90% CI for RMSEA	Interval does not contain 0.1	[0.042, 0.050]	Good
SRMR (Standardized Root Mean Square Residual)	< 0.08	0.043	Excellent

PPLI, Perceived Physical Literacy; RAI, Relative Autonomy Index for Autonomous Motivation; SWB, Subjective Wellbeing. All coefficients were obtained after controlling for gender, age, and BMI.

**FIGURE 4:**
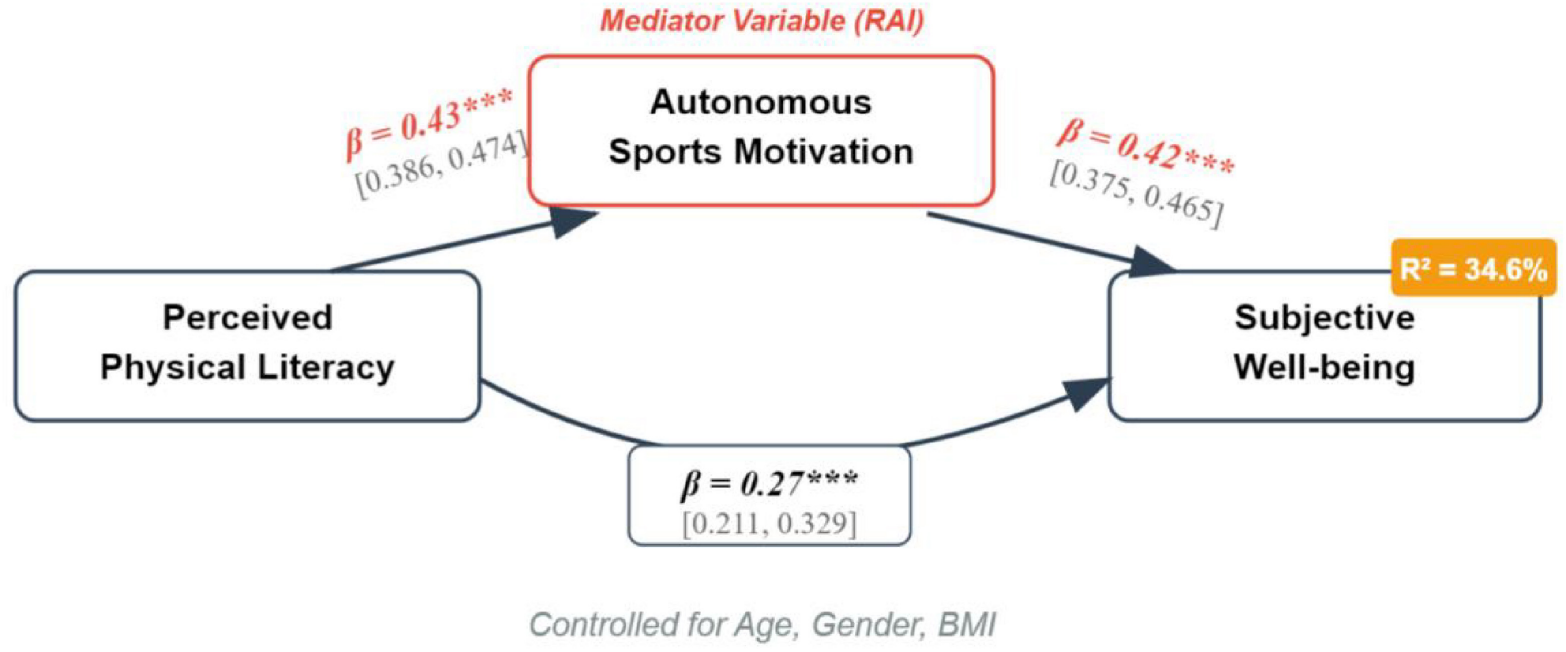
Structural path model of the indirect association of autonomous sports motivation (RAI) on subjective wellbeing. *N* = 858. β represents the standardized path coefficient, followed by its 95% confidence interval in brackets. ***p < 0.001. Model estimates controlled for gender, age, and BMI.

The final mediation effect model structure and standardized path results are shown in Figure 4. The model accounted for a substantial portion of the variance in subjective wellbeing (*R*^2^ = 0.35), demonstrating considerable explanatory power. It is important to note that the use of RAI as a single-score mediator was empirically justified by the high correlation between the RAI and the autonomous motivation latent construct (reported in section 3.3), ensuring that the structural model remains parsimonious without losing the nuance of motivational quality.

#### 3.4.1 Path coefficient analysis

The specific path analysis results (see the upper part of Figure 4) strongly supported the initial hypotheses of this study:

Path a (PPLI → RAI): Perceived physical literacy had a significant association with autonomous sports motivation (β = 0.43, *p* < 0.001, 95%CI [0.386, 0.474]), validating hypothesis H2 and indicating that physical literacy is a key antecedent for igniting intrinsic motivation for participation.

Path b (RAI → SWB): Autonomous sports motivation had a significant positive association with subjective wellbeing (β = 0.42, *p* < 0.001, 95%CI [0.375, 0.465]), validating hypothesis H3.

Direct Path c’ (PPLI → SWB): After including the mediator variable, physical literacy still maintained a significant direct association with wellbeing (β = 0.27, *p* < 0.001, 95%CI [0.211, 0.329]), validating hypothesis H1.

#### 3.4.2 Mediation effect test and decomposition

This study used a bias-corrected Bootstrap procedure (5000 resamples) to rigorously test the mediation effect (see Table 6). The results showed that the unstandardized indirect effect of autonomous sports motivation was 0.233, with a 95% confidence interval of 0.186,0.281 (standardized effect β = 0.18). Since this interval does not contain zero (see the CI forest plot at the bottom of Figure 4), it confirms that autonomous sports motivation plays a significant partial mediating role in the relationship, fully supporting hypothesis H4.

**TABLE 6:** Test results of indirect association path coefficients.

Effect path	Symbol	Path description	Unstandardized (B)	Standard error (SE)	Standardized (β)	95% CI	*p*-value
Direct effect	c’	PPLI → SWB	0.354	0.040	0.27	[0.211, 0.329]	<0.001
Indirect effect	a*b	PPLI → RAI → SWB	0.233	0.024	0.18	[0.186, 0.281]	<0.001
Mediation path a	a	PPLI → RAI	3.945	0.282	0.43	[0.386, 0.474]	<0.001
Mediation path b	b	RAI → SWB	0.059	0.004	0.42	[0.375, 0.465]	<0.001
Total effect	c	PPLI → SWB	0.587	0.040	0.45	[0.392, 0.508]	<0.001
Proportion mediated		(a*b)/c	39.7%				
Model *R*^2^		SWB	0.35				

95% CI refers to the bias-corrected bootstrap confidence interval based on 5,000 resamples. RAI, Relative Autonomy Index.

In terms of variance explained (as detailed in Table 6), 39.7% of the total association of physical literacy on wellbeing was indirectly realized by stimulating students’ autonomous motivation. This finding highlights that “motivation internalization” is an indispensable bridge for transforming physical functioning into psychological wellbeing.

### 3.5 Multi-group comparison of the mediation model across different exercise habits

To further explore whether the “physical literacy-autonomous motivation-wellbeing” mediation mechanism proposed in this study has broad generalizability across students with different extracurricular exercise habits, this study introduced participants’ usual extracurricular exercise frequency as a boundary condition for multi-group analysis. This analysis aimed to explore whether the association between physical literacy and psychological wellbeing was comparable for students with different levels of extracurricular exercise habits. This question has been rarely addressed in previous literature (Donnelly et al., 2024), and the multi-group design of this study provides preliminary empirical support.

This study employed a multi-group structural equation modeling (Multi-Group SEM) approach. Based on the predefined grouping criterion, the sample was divided into a “low-to-moderate frequency group” (exercising 2 times a week or less, predominantly insufficient activity, *n* = 551) and a “high-frequency group” (exercising 3 times a week or more, *n* = 307). Prior to the structural path comparison, a baseline configural multi-group measurement model exhibited an acceptable fit to the data (χ^2^/*df* = 2.45, CFI = 0.938, RMSEA = 0.041), providing preliminary support for comparing structural paths across the two groups. Subsequently, we estimated the parameters of the core model paths for both groups and used the *Z*-test to compare the path coefficients across groups. The results are shown in Table 7.

**TABLE 7:** Multi-group comparison results for extracurricular exercise frequency.

Path	Low-frequency group (*n* = 551) Standardized β	High-frequency group (*n* = 307) Standardized β	Difference test *Z*-value	*p*-value	Conclusion
Path a (PPLI → RAI)	0.44***	0.43***	−0.03	0.973	Invariant across groups
Path b (RAI → SWB)	0.39***	0.46***	1.33	0.183	Invariant across groups
Path c’ (PPLI →SWB)	0.30***	0.23***	−1.06	0.290	Invariant across groups

*β-*values are standardized coefficients after controlling for gender and age; ***p < 0.001.

The data revealed a finding of great practical significance: the facilitative association of perceived physical literacy with sports motivation (path *a*: *Z* = −0.03, *p* = 0.973) and the association of sports motivation with subjective wellbeing (path *b*: *Z* = 1.33, *p* = 0.183) showed no statistically significant differences between the two groups. This suggests that the association pattern between physical literacy and wellbeing was similar across the two exercise-frequency groups that is independent of the students’ current exercise levels.

**FIGURE 5:**
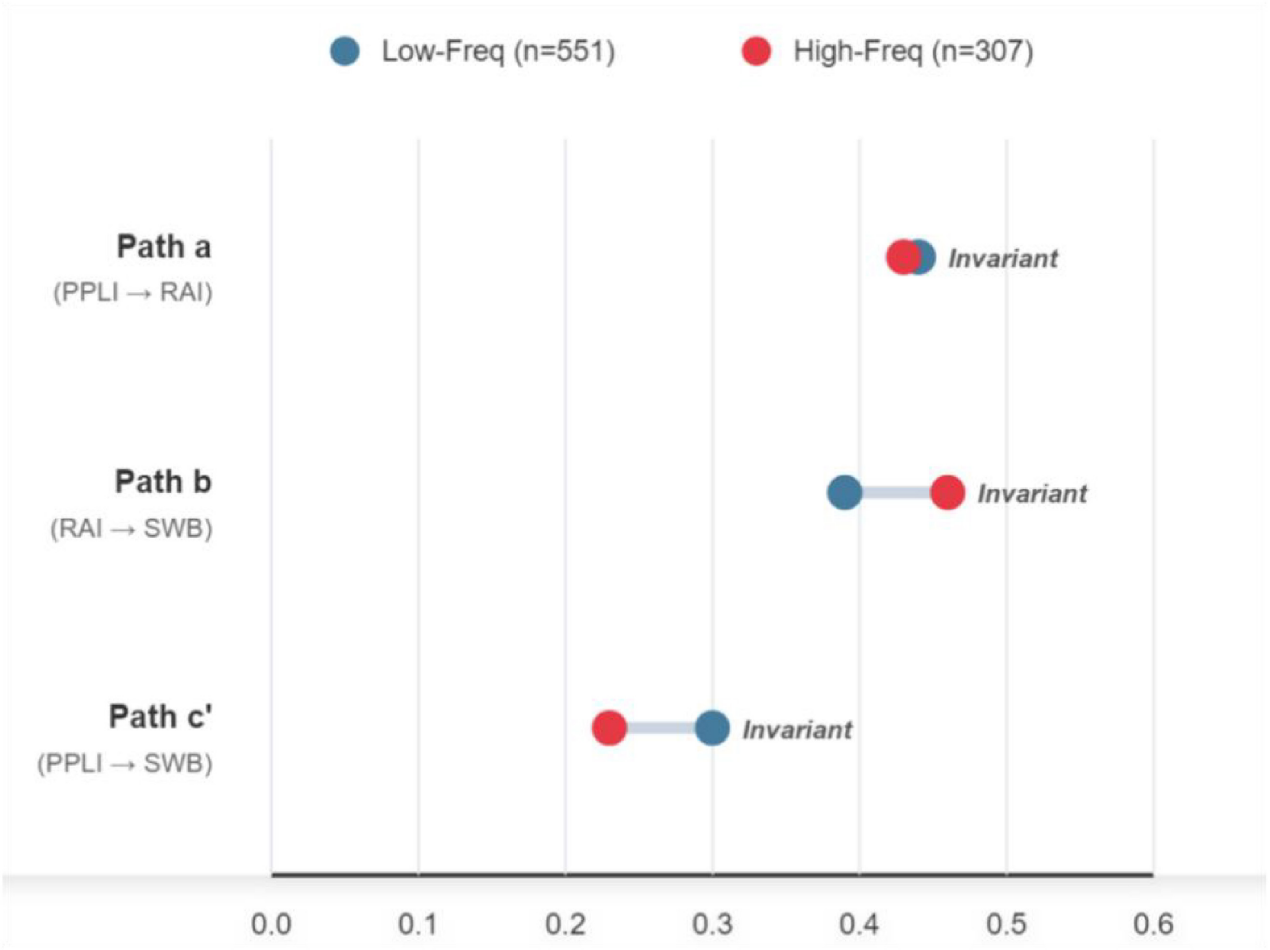
Comparison of standardized path coefficients between groups with different extracurricular exercise frequencies. Low-frequency *n* = 551; High-frequency *n* = 307. The scatter points represent the standardized path coefficients β for the low-frequency and high-frequency groups, respectively. The points for each path are extremely close, visually indicating that the model paths were broadly similar across groups in the multi-group analysis.

As illustrated in Figure 5, we visually suggests this cross-group robustness through a coefficient distribution comparison plot. The standardized coefficients of the core paths are highly convergent across different groups, with the scatter points showing a tight distribution along the horizontal axis. This provides preliminary support for the generalizability of the mediation model: regardless of whether students have long-term exercise habits outside of class, higher perceived physical literacy in PE contexts is associated with higher-quality autonomous motivation for sports, thereby significantly improving their subjective wellbeing experience (Liu et al., 2024). This finding further supports the theoretical relevance of physical literacy as a core educational goal that transcends behavioral baselines (Gao et al., 2024).

## 4 Discussion

### 4.1 Overview and interpretation of findings

This study systematically examined the complex relationships among students’ perceived physical literacy, autonomous sports motivation, and subjective wellbeing in the context of university physical education. The results suggest an indirect statistical association linking “physical competence” to psychological health outcomes: perceived physical literacy was positively associated with subjective wellbeing, and this association was also observed indirectly through autonomous sports motivation, which may help explain part of the relationship. Specifically, when students feel more competent, knowledgeable, and confident in physical activities, their reasons for participating become more internal and autonomous. This more internalized form of motivation was associated with greater life satisfaction and emotional vitality (Diener et al., 1985; Ryan and Frederick, 1997). This finding not only aligns with all our proposed hypotheses but also illustrates the intrinsic logic of the psychological benefits of physical education.

### 4.2 Theoretical implications and dialogue with literature

The findings of this study contribute to the theoretical literature in at least three ways.

First, it significantly enriches the understanding of Physical Literacy (PL) theory. Previous research has largely focused on the association of physical literacy on physical health indicators or physical activity participation behaviors (Sum et al., 2016; Luo et al., 2022; Liu et al., 2024). This study suggests that physical literacy may not only be related to physical competence but may also be linked to psychological resources that are robustly associated with positive psychological experiences (Ma et al., 2021). It may extend the research perspective of PL theory from behavioral science to the field of positive psychology, echoing the research directions of Melby et al. (2023), Souza-Lima et al. (2025), and Gao et al. (2024), and collectively pushing the theoretical boundaries of the field.

Second, this study provides a vivid empirical case for the application of Self-Determination Theory (SDT) in a specific educational context. The core of SDT lies in satisfying basic psychological needs to promote motivation internalization (Vallerand et al., 2008; Ntoumanis et al., 2021). Our research specifies that the “cultivation of physical literacy” serves as an environment satisfying students’ needs for “competence” and “autonomy” in PE classes (Abdulla et al., 2022; Tilga et al., 2023). It grounds the theoretical logic of SDT, connecting it closely with the concrete content of physical education teaching, and provides evidence for how PE classes relate to motivational quality (Hutmacher et al., 2022; Llanos-Muñoz et al., 2023). Furthermore, this study clarifies the incremental contribution over findings from Ma et al. (2021) and Melby et al. (2023). While Ma et al. (2021) established the link between PL and mental health components like resilience, and Melby et al. (2023) emphasized the role of activity participation, our results suggest that the psychological internalization of motivation (the RAI pathway) may be an important explanatory link for long-term health. In the specific context of Chinese university physical education—characterized by mandatory attendance and a collectivist educational culture—enhancing physical literacy serves as a cognitive buffer. It may help students reinterpret institutional requirements as opportunities for a more self-determined pursuit of “proactive health,” ensuring that even compulsory participation translates into an experience of wellbeing.

Finally, the findings of this study also align with the theoretical trend of “Embodied Cognition” (Sauri et al., 2025). To strengthen this theoretical link, our results demonstrate that a higher cognitive evaluation of bodily competence (physical literacy) is associated with enhanced energetic psychological states (subjective vitality, as measured by Bostic et al., 2000). This provides empirical evidence that wellbeing perceptions are not isolated from physical self-perceptions. It suggests that the body is not merely a vessel for the mind but an inseparable component of the mental experience itself.

### 4.3 Practical implications: from “teaching techniques” to “cultivating motivation”

These theoretical discussions must ultimately return to how we can adjust our educational practices. The associations observed in this study offer preliminary guidance for the reform of university physical education in China and globally (Castellote-Caballero et al., 2024; Herbert, 2022). A key implication is that the goal of physical education may be broadened: from a singular emphasis on “teaching techniques and meeting standards” to a greater focus on “nurturing literacy and promoting motivation.”

For frontline physical educators, this means a suggested shift in teaching strategies. The focus of the class may be shifted away from uniform skill drills and tedious strength training, but on becoming a “guide” who can stimulate students’ intrinsic enjoyment and cultivate their lifelong awareness of physical activity (Tilga et al., 2023; Llanos-Muñoz et al., 2023). For example, teachers can: (1) Provide choice and autonomy: Within the curriculum framework, allow students to choose different sports or exercise methods based on their interests and physical condition; (2) Emphasize process and progress through constructive feedback (Haug et al., 2023; Lunde et al., 2023); (3) Create connection and funthrough collaborative teaching activities (Abdulla et al., 2022). Despite these implications, implementation barriers exist. Current university evaluation systems often prioritize standardized fitness scores, which as our results suggest, may limit the development of autonomy.

For university curriculum designers and administrators, the research findings suggest a potential direction for reform (Hu and Wang, 2026). The results indicate that offering diverse options (e.g., yoga, rock climbing) might align with diverse student needs, facilitating the internalization of their motivation (Jiang et al., 2024). A possible goal is to shift PE classes from a course students “have to take” into a supportive environment that where students can draw energy and adopt a sustainable healthy lifestyle.

Furthermore, the supplementary multi-group analysis in this study provides a strong boost of confidence for teaching “beginners” or “exercise-resistant” groups. Our data show that the association strength between physical literacy and wellbeing via autonomous motivation was consistent across students’ past exercise habits (the association strength did not differ across groups). This resonates with the findings of Qi et al. (2024) and de Oliveira Barbosa et al. (2024). This implies that for “sedentary” students educators who cultivate their sense of physical competence and knowledge may help ignite intrinsic motivation (Donnelly et al., 2024). This also suggests that compulsory university PE should not become a club for a few sports enthusiasts, but rather an essential educational platform to engage these “marginalized exercisers” and achieve equity in physical and mental health.

### 4.4 Limitations and future directions

First, the cross-sectional design of this study inherently limits our ability to establish strict causality, allowing us only to reveal associations between variables. Although our theoretical model is grounded in motivational theories and suggests a plausible directional framework (Vallerand et al., 2008; Ntoumanis et al., 2021) and has a clear causal logic, we cannot rule out the possibility of reverse causality (e.g., students with higher wellbeing might be more willing to participate in physical activities, thereby enhancing their physical literacy). Therefore, future research would greatly benefit from adopting longitudinal or cross-lagged panel designs, collecting data at multiple time points to more reliably test the dynamic causal relationships between variables (Melby et al., 2023).

Second, the data were entirely dependent on participants’ self-report questionnaires, which introduces the potential risk of common method variance (CMV). Although the Harman’s single-factor test in our study indicated that CMV was not a serious issue, and we tried to mitigate it through procedures like anonymous responses, uncontrollable factors such as social desirability bias (i.e., students may tend to report more positive attitudes) or personal emotional states could still have a subtle influence on data precision (Jiang et al., 2024). Future research that incorporates objective measurement indicators (e.g., actual exercise duration recorded by wearable devices, physical competence data from fitness tests) would yield more robust conclusions (Ahsan and Abualait, 2025).

Third, while our sample size was adequate, it was primarily drawn from universities in mainland China. Given the unique collectivist culture and compulsory physical education requirements (credits) in this context, the conclusions of this study should be generalized to other cultures (especially Western individualistic cultures) with caution (Mendoza-Muñoz et al., 2023; Gandrieau et al., 2023). For example, under China’s unique collectivist culture and “compulsory PE credit” policy, the process of students’ transformation from “external motivation to internalized motivation” may have its own local characteristics (Jiang et al., 2024; Hu and Wang, 2026). Cross-cultural comparative research would be a very valuable direction for future exploration.

Finally, more than half of the variance in wellbeing remains primarily by other factors outside the current model. Variables such as students’ individual personality traits (e.g., extraversion, neuroticism), family socioeconomic status (SES), social support networks, and recent major life events could all be important correlates of wellbeing (Moussa-Chamari et al., 2024; Talapko et al., 2021). Future research could attempt to build more complex integrated models that include these factors to gain a more comprehensive understanding of the sources of wellbeing among university students (Sarfo et al., 2023).

## 5 Conclusion

Through an empirical investigation of 858 Chinese university students, this study identifies a positive association between perceived physical literacy and subjective wellbeing and indicates a significant indirect path via autonomous sports motivation. The findings strongly suggest that the value of university physical education relates to more than just shaping students’ healthy bodies but, more importantly, in cultivating their intrinsic and sustainable willingness to lead a healthy lifestyle (Ntoumanis et al., 2021; Sauri et al., 2025). The findings suggest that when students feel more competent, more confident, and more knowledgeable about sports (Ma et al., 2020; Luo et al., 2022), they may be more likely to develop intrinsic enjoyment of physical activity (Vallerand et al., 2008; Tilga et al., 2023), which in turn is associated with greater wellbeing (Diener et al., 1985; Ryan and Frederick, 1997; Bostic et al., 2000). Therefore, promoting a paradigm shift in physical education from a “skill-based” to a “literacy and motivation-based” approach is a relevant direction for future education reformers and practitioners (Hu and Wang, 2026; Gao et al., 2024; Liu et al., 2024).

## Data Availability

The original contributions presented in this study are included in the article/Supplementary material, further inquiries can be directed to the corresponding author.

## References

[B1] Abdulla A. Whipp P. R. McSporran G. Teo T. (2022). An interventional study with the Maldives generalist teachers in primary school physical education: An application of self-determination theory. *PLoS One* 17:e0268098. 10.1371/journal.pone.0268098 35522650 PMC9075671

[B2] Ahsan M. Abualait T. (2025). Investigation of the relationship between mental health and physical activity among university students. *Front. Psychol.* 15:1546002. 10.3389/fpsyg.2024.1546002 39839918 PMC11747765

[B3] Anderson J. C. Gerbing D. W. (1988). Structural equation modeling in practice: A review and recommended two-step approach. *Psychol. Bull.* 103:411. 10.1037/0033-2909.103.3.411

[B4] Auerbach R. P. Mortier P. Bruffaerts R. Alonso J. Benjet C. Cuijpers P. et al. (2018). WHO world mental health surveys international college student project: Prevalence and distribution of mental disorders. *J. Abnormal Psychol.* 127:623. 10.1037/abn0000362 30211576 PMC6193834

[B5] Bostic T. J. McGartland Rubio D. Hood M. (2000). A validation of the subjective vitality scale using structural equation modeling. *Soc. Indicators Res.* 52 313–324. 10.1023/A:1007136110218

[B6] Castellote-Caballero Y. Carcelén-Fraile M. D. C. Aibar-Almazán A. Rivas-Campo Y. González-Martín A. M. (2024). Yoga as a therapeutic approach to mental health in university students: A randomized controlled trial. *Front. Public Health* 12:1406937. 10.3389/fpubh.2024.1406937 38903593 PMC11188441

[B7] Chautrakarn S. Jaiprom E. Ong-Artborirak P. (2024). Mental health and sleep in the post-COVID-19 era among Thai undergraduate students. *Sci. Rep.* 14:26584. 10.1038/s41598-024-78559-0 39496814 PMC11535199

[B8] de Oliveira Barbosa R. Castilho, dos Santos G. da Silva J. M. de Souza Silva T. M. Dias P. H. G. et al. (2024). Does autonomous motivation and self-efficacy mediate associations between environmental factors and physical activity in adolescents? *BMC Psychol.* 12:548. 10.1186/s40359-024-02055-3 39394162 PMC11468100

[B9] Deci E. L. Ryan R. M. (2000). The” what” and” why” of goal pursuits: Human needs and the self-determination of behavior. *Psychol. Inquiry* 11 227–268. 10.1207/S15327965PLI1104_01

[B10] Diener E. D. Emmons R. Larsen R. Griffin S. (1985). The satisfaction with life scale. *J. Personality Assessment* 49 71–75. 10.1207/s15327752jpa4901_13 16367493

[B11] Donnelly S. Penny K. Kynn M. (2024). The effectiveness of physical activity interventions in improving higher education students’ mental health: A systematic review. *Health Promot. Int.* 39:daae027. 10.1093/heapro/daae027 38563387 PMC10985680

[B12] Gandrieau J. Dieu O. Potdevin F. Derigny T. Schnitzler C. (2023). Measuring physical literacy for an evidence-based approach: Validation of the French perceived physical literacy instrument for emerging adults. *J. Exerc. Sci. Fitness* 21 295–303. 10.1016/j.jesf.2023.06.001 37520158 PMC10373918

[B13] Gao T. Y. Huang F. H. Liu T. Sum R. K. W. De Liu J. Tang D. et al. (2024). The role of physical literacy and mindfulness on health-related quality of life among college students during the COVID-19 pandemic. *Sci. Rep.* 14:237. 10.1038/s41598-023-50958-9 38167897 PMC10761947

[B14] Haug E. Castillo I. Samdal O. Smith O. R. F. (2023). Body-related concerns and participation in physical education among adolescent students: The mediating role of motivation. *Front. Psychol.* 14:1266740. 10.3389/fpsyg.2023.1266740 37842720 PMC10569498

[B15] Herbert C. (2022). Enhancing mental health, well-being and active lifestyles of university students by means of physical activity and exercise research programs. *Front. Public Health* 10:849093. 10.3389/fpubh.2022.849093 35548074 PMC9082407

[B16] Hu R. Wang W. (2026). A structural model of perceived physical literacy of educational/sports college students based on PPLI. *Sci. Rep.* 16:2955. 10.1038/s41598-025-32834-w 41453960 PMC12830928

[B17] Hutmacher D. Eckelt M. Bund A. Melzer A. Steffgen G. (2022). Uncovering the role of mindfulness in autonomous motivation across physical education and leisure time: Extending the trans-contextual model. *Int. J. Environ. Res. Public Health* 19:12999. 10.3390/ijerph192012999 36293586 PMC9603215

[B18] Jankauskiene R. Urmanavicius D. Baceviciene M. (2022). Association between motivation in physical education and positive body image: Mediating and moderating effects of physical activity habits. *Int. J. Environ. Res. Public Health* 20:464. 10.3390/ijerph20010464 36612785 PMC9819534

[B19] Jiang S. Ng J. Y. Choi S. M. Ha A. S. (2024). Relationships among eHealth literacy, physical literacy, and physical activity in Chinese university students: Cross-sectional study. *J. Med. Internet Res.* 26:e56386. 10.2196/56386 39496161 PMC11574492

[B20] Liu C. Y. Lin L. L. C. Sheu J. J. Sum R. K. W. (2022). Psychometric validation of senior perceived physical literacy instrument. *Int. J. Environ. Res. Public Health* 19:6726. 10.3390/ijerph19116726 35682309 PMC9179957

[B21] Liu Y. Liu S. X. Sum R. K. W. Duncan M. J. Gu Y. D. Li M. H. (2024). Associations between levels of physical literacy and adherence to the 24-h movement guidelines among university students: A cross-sectional study. *J. Exerc. Sci. Fitness* 22 221–226. 10.1016/j.jesf.2024.03.006 38559907 PMC10979097

[B22] Llanos-Muñoz R. Vaquero-Solís M. López-Gajardo M. Á Sánchez-Miguel P. A. Tapia-Serrano M. Á Leo F. M. (2023). Intervention programme based on self-determination theory to promote extracurricular physical activity through physical education in primary school: A study protocol. *Children* 10:504. 10.3390/children10030504 36980062 PMC10047147

[B23] Lunde C. Reinholdsson T. Skoog T. (2023). Unexcused absence from physical education in elementary school. On the role of autonomous motivation and body image factors. *Body Image* 45 229–237. 10.1016/j.bodyim.2023.03.007 36965234

[B24] Luo L. Song N. Huang J. Zou X. Yuan J. Li C. et al. (2022). Validity evaluation of the college student physical literacy questionnaire. *Front. Public Health* 10:856659. 10.3389/fpubh.2022.856659 35692349 PMC9178231

[B25] Ma R. S. Sum R. K. Hu Y. N. Gao T. Y. (2020). Assessing factor structure of the simplified Chinese version of perceived physical literacy instrument for undergraduates in Mainland China. *J. Exerc. Sci. Fitness* 18 68–73. 10.1016/j.jesf.2020.01.001 31998384 PMC6965736

[B26] Ma R. Liu T. Raymond Sum K. W. Gao T. Li M. Choi S. M. et al. (2021). Relationship among physical literacy, mental health, and resilience in college students. *Front. Psychiatry* 12:767804. 10.3389/fpsyt.2021.767804 34966305 PMC8710533

[B27] Markland D. Tobin V. (2004). A modification to the behavioural regulation in exercise questionnaire to include an assessment of amotivation. *J. Sport Exerc. Psychol.* 26 191–196. 10.1123/jsep.26.2.191

[B28] Melby P. S. Elsborg P. Bentsen P. Nielsen G. (2023). Cross-sectional associations between adolescents’ physical literacy, sport and exercise participation, and wellbeing. *Front. Public Health* 10:1054482. 10.3389/fpubh.2022.1054482 36926143 PMC10011712

[B29] Mendoza-Muñoz M. Carlos-Vivas J. Castillo-Paredes A. Sum R. K. W. Rojo-Ramos J. Pastor-Cisneros R. (2023). Translation, cultural adaptation and validation of perceived physical literacy instrument-Spanish version (PPLI-Sp) for adults. *Journal Sports Sci. Med.* 22:455. 10.52082/jssm.2023.455 37711722 PMC10499136

[B30] Moussa-Chamari I. Farooq A. Romdhani M. Washif J. A. Bakare U. Helmy M. et al. (2024). The relationship between quality of life, sleep quality, mental health, and physical activity in an international sample of college students: A structural equation modeling approach. *Front. Public Health* 12:1397924. 10.3389/fpubh.2024.1397924 39050600 PMC11266085

[B31] Ntoumanis N. Ng J. Y. Prestwich A. Quested E. Hancox J. E. Thøgersen-Ntoumani C. et al. (2021). A meta-analysis of self-determination theory-informed intervention studies in the health domain: Effects on motivation, health behavior, physical, and psychological health. *Health Psychol. Rev.* 15 214–244. 10.1080/17437199.2020.1718529 31983293

[B32] Podsakoff P. M. MacKenzie S. B. Lee J. Y. Podsakoff N. P. (2003). Common method biases in behavioral research: A critical review of the literature and recommended remedies. *J. Appl. Psychol.* 88:879. 10.1037/0021-9010.88.5.879 14516251

[B33] Qi Y. Yin Y. Wang X. Zou Y. Liu B. (2024). Autonomous motivation, social support, and physical activity in school children: Moderating effects of school-based rope skipping sports participation. *Front. Public Health* 12:1295924. 10.3389/fpubh.2024.1295924 38327571 PMC10847259

[B34] Rodríguez-Romo G. Acebes-Sánchez J. García-Merino S. Garrido-Muñoz M. Blanco-García C. Diez-Vega I. (2022). Physical activity and mental health in undergraduate students. *Int. J. Environ. Res. Public Health* 20:195. 10.3390/ijerph20010195 36612516 PMC9819335

[B35] Ryan R. M. Frederick C. (1997). On energy, personality, and health: Subjective vitality as a dynamic reflection of well-being. *J. Pers.* 65 529–565. 10.1111/j.1467-6494.1997.tb00326.x 9327588

[B36] Sarfo J. O. Obeng P. Kyereh H. K. Ansah E. W. Attafuah P. Y. A. (2023). Self-determination theory and quality of life of adults with diabetes: A scoping review. *J. Diabetes Res.* 2023:5341656. 10.1155/2023/5341656 37091043 PMC10115521

[B37] Sauri S. Subarjah H. Firdaus F. S. Efendi T. (2025). Building mental well-being through physical literacy: The role of physical activity from an embodied cognition perspective. *Active J. Phys. Educ. Sport Health Recreation* 14 839–845. 10.15294/active.v14i3.34234

[B38] Souza-Lima J. D. Ortiz-Marholz P. Ferrari G. Parra-Saldias M. Duclos-Bastias D. Godoy-Cumillaf A. et al. (2025). Machine learning-mediated analysis of physical literacy in children’s subjective well-being: Evidence from a multinational survey. *Psychiatry Int.* 6:131. 10.3390/psychiatryint6040131

[B39] Sum R. K. W. Ha A. S. C. Cheng C. F. Chung P. K. Yiu K. T. C. Kuo C. C. et al. (2016). Construction and validation of a perceived physical literacy instrument for physical education teachers. *PLoS One* 11:e0155610. 10.1371/journal.pone.0155610 27195664 PMC4873233

[B40] Talapko J. Perić I. Vulić P. Pustijanac E. Jukić M. Bekić S. et al. (2021). Mental health and physical activity in health-related university students during the COVID-19 pandemic. *Healthcare* 9:801. 10.3390/healthcare9070801 34202384 PMC8304952

[B41] Tilga H. Vahtra K. Koka A. (2023). The role of teachers (de-) motivational styles on students’ autonomous motivation in physical education and leisure time. *Baltic J. Health Phys. Activity* 15:5. 10.29359/BJHPA.15.4.05

[B42] Vallerand R. J. Pelletier L. G. Koestner R. (2008). Reflections on self-determination theory. *Canadian Psychol.* 49:257. 10.1037/a0012804

[B43] Whitehead M. (2010). *Physical Literacy: Throughout the Lifecourse.* Milton Park: Routledge.

[B44] Wilson P. M. Rodgers W. M. Loitz C. C. Scime G. (2006). “It’s Who I Am… Really!” The importance of integrated regulation in exercise contexts. *J. Appl. Biobehav. Res.* 11 79–104. 10.1111/j.1751-9861.2006.tb00021.x

