# Supplementary material for: Perceived physical literacy and subjective wellbeing in university students: the indirect association via autonomous sports motivation
Source: Front Psychol. 2026 Jul 10;17:1860366. doi: 10.3389/fpsyg.2026.1860366 (PMC13395672; doi:10.3389/fpsyg.2026.1860366)
Supplement: Supplementary file 1 [file Data_Sheet_1.zip › Supplementary_Material.docx]

**Appendix A: English Version of the Survey Instrument**

The survey was originally administered in simplified Chinese to ensure accurate linguistic and cultural comprehension among the target population, i.e., mainland Chinese university students. The established scales utilized in this study were translated and cross-checked based on their original English versions. The following is the English counterpart of the survey.

Survey on University Students’ Sports Participation and Mental Health Experience

Introduction and Informed Consent

Dear student,
We are a joint research team focusing on university sports and mental health. We are currently conducting an academic survey to understand university students’ experiences in physical education (PE) classes, their exercise habits, and how these are associated with their daily lives.
This questionnaire will take about 5 minutes to complete. Your responses are vital to our research. This survey is strictly anonymous. All data collected will be used solely for aggregate statistical analysis and academic publications. Your raw data will be securely encrypted, and no personal privacy will be leaked. Your participation is entirely voluntary, and you may withdraw from the survey at any time.
Due to ethical committee regulations, this study is restricted to university students aged 18 and above.

**1. Are you 18 years of age or older, and do you voluntarily agree to participate in this survey?**

Yes, I am at least 18 years old and consent to participate. (Proceed to Question 2)

No. (End of survey, thank you for your time)

**Part 1: Basic Information and Exercise Habits**

**2. Your Gender:**

Male

Female
**3. Your Actual Age:** ______ years
**4. Your Current Grade Level:**

Freshman

Sophomore

Junior

Senior

Postgraduate
**5. Your Major Discipline:**

Science, Engineering, Agriculture, or Medicine

Humanities, History, Philosophy, Education, or Law

Economics or Management

Arts or Physical Education
**6. Are you currently enrolled in a mandatory university Physical Education (PE) course?**

Yes, currently enrolled this semester

No, have completed the mandatory PE credits
**7. Your Height (cm):** ______
**8. Your Weight (kg):** ______
**9. Overall, excluding short-term factors like catching a cold, how would you evaluate your current general physical health?**

**Very poor**

Poor

Fair

Good

Very good
**10. Excluding regular university PE classes, what is your average frequency of extracurricular physical exercise (moderate intensity, i.e., slight sweating and faster breathing) per week?**

Rarely or never

1-2 times per week

3-4 times per week

5 or more times per week

**Part 2: Self-Perception and Physical Competence Experience (PPLI)**

Instructions: The following statements describe your views on your physical abilities and the value of sports. Please read each carefully and select the option that best reflects your actual situation (1 = Strongly disagree, 2 = Disagree, 3 = Neutral, 4 = Agree, 5 = Strongly agree).

11. I possess the fundamental movement skills for regular daily physical activities.
12. I have enough physical strength and endurance to meet the demands of university PE classes and daily exercises.
13. I have confidence to try and learn new challenging sports activities.
14. I am aware of the specific benefits of different types of physical activities for maintaining physical and mental health.
15. I can apply the sports knowledge learned in PE classes or from coaches to my daily exercise routines.
16. I believe that maintaining an active physical state throughout my life is essential for my holistic development.
17. I am able to acutely sense and adjust my physical state (e.g., heart rate, muscle fatigue) during exercise.
18. I can communicate and cooperate well with peers or participants during PE classes or team sports.
19. (Attention Check) For this attention check question, to ensure we are not analyzing automated responses, please directly select “Disagree” (2).

**Part 3: Drivers of Sports Participation (BREQ-3)**

Instructions: People participate in PE classes or extracurricular sports for different reasons. To what extent do the following statements correspond to your true reasons for participating in physical activities (1 = Not true for me, 2 = Slightly true for me, 3 = Moderately true for me, 4 = Very true for me, 5 = Completely true for me).

20.I don’t see why I should have to exercise, I think it’s a waste of time.
21. I exercise because my family/friends advise it, or to meet university credit requirements.
22. I would feel guilty or blame myself regarding my physical state if I didn’t exercise regularly.
23. I think regular exercise is highly beneficial for my health, and this is something I rationally value.
24. Regular physical exercise has become an indispensable part of my lifestyle principles.
25. Participating in physical activities makes me feel very happy, relaxed, and enjoying myself.
26. I would feel like someone with bad lifestyle habits if I didn’t exercise.
27. I consider exercise a necessary part of maintaining a healthy lifestyle.
28. I consider myself fundamentally a “sports-loving person”; this is part of my self-identity.
29. Sweating and mastering new skills during exercise makes me feel full of fun.
30. I can’t see any practical meaning in exercising, and I don’t want to participate at all.

**Part 4: Recent Life Experience Scale (SWB)**

Instructions: Sports participation often subtly affects our life status. Below are statements describing your overall life perception and energy status over the past month. Please check the number that best fits your feelings (1 = Strongly disagree, 2 = Disagree, 3 = Slightly disagree, 4 = Neither agree nor disagree, 5 = Slightly agree, 6 = Agree, 7 = Strongly agree).

31. In most ways my life is close to my ideal.
32. Overall, I am very satisfied with my university life and current status.
33. So far I have gotten the important things I want in life.
34. If I could live my life over, I would change almost nothing.
35. Recently, I feel full of energy and vitality.
36. I feel that my inner self is full of life and energetic.
37. I look forward to the arrival of a new day and can always face life with enthusiasm.
38. I rarely feel completely mentally exhausted; I am refreshed most of the time.

**Appendix B: Data Codebook and Scoring Key**

To facilitate reproducibility, the following table summarizes the data structure and scoring methods applied in our mediation analysis.

| Construct / Variable | Origin Items (Survey Q#) | Coding & Calculation Method |
| --- | --- | --- |
| **I. Control Variables** |  |  |
| Gender | Q2 | 1 = Male, 2 = Female |
| Age | Q3 | Continuous |
| Grade | Q4 | 1 = Freshman to 5 = Postgraduate |
| BMI | Q7 & Q8 | Computed: Weight(kg) / (Height(m))^2 |
| Health Status | Q9 | Ordinal: 1 (Very poor) to 5 (Very good) |
| Ex_Frequency | Q10 | Ordinal: 1 to 4 |
| **II. Subscales & Mediators** |  |  |
| Physical Literacy (PPLI) | Q11 - Q18 | Computed: Mean of Q11 to Q18 (1-5 Likert) |
| Attention Check | Q19 | If score ≠ 2, respondent was excluded |
| Sports Motivation (BREQ-3) | Q20 - Q30 | Calculated using the Relative Autonomy Index (RAI) |
| - Amotivation | Q20, Q30 | Mean of items |
| - External Regulation | Q21 | Mean of items |
| - Introjected Regulation | Q22, Q26 | Mean of items |
| - Identified Regulation | Q23, Q27 | Mean of items |
| - Integrated Regulation | Q24, Q28 | Mean of items |
| - Intrinsic Regulation | Q25, Q29 | Mean of items |
| RAI Score (Mediator) | Above 6 dimensions | See the calculation formula below |
| **III. Dependent Variable** |  |  |
| Subjective Well-Being (SWB) | Q31 - Q38 | Computed: Mean of all 8 items (1-7 Likert) |
| - Cognitive Dimension (SWLS) | Q31 - Q34 | Satisfaction with Life Scale components |
| - Affective Dimension (SVS) | Q35 - Q38 | Subjective Vitality Scale components |

### Scoring Key for Motivation (RAI Formulation):

In this study, the overall quality of sports motivation was calculated using the Relative Autonomy Index (RAI). Based on the Self-Determination Theory (Vallerand et al., 2008), the subscales were universally weighted and summed to form a single continuous index for the mediation analysis. A higher RAI score indicates a more autonomous, self-determined motivation profile.

Using the computed subscale mean scores, the formula applied is:

RAI = (-3 × Amotivation) + (-2 × External) + (-1 × Introjected) + (1 × Identified) + (2 × Integrated) + (3 × Intrinsic)

Note: Data points failing the attention check item [Q19] were removed listwise prior to descriptive and inferential statistics.
